# Reoperation for ipsilateral local recurrence following prior nephron-sparing surgery: innovative surgical insights from a high-volume urological center with cross-sectional study

**DOI:** 10.1097/JS9.0000000000001917

**Published:** 2024-07-02

**Authors:** Wenming Ma, Wenlong Xu, Jialin Meng, Lei Chen, Sheng Tai, Cheng Yang, Jinhu Chen, Haoqiang Shi, Chaozhao Liang

**Affiliations:** a Department of Urology, The First Affiliated Hospital of Anhui Medical University; b Institute of Urology, Anhui Medical University; c Anhui Province Key Laboratory of Urological and Andrological Diseases Research and Medical Transformation; d Center for Big Data and Population Health of IHM, Hefei, People’s Republic of China

**Keywords:** local recurrence, nephron-sparing surgery, reoperation, surgical strategies

## Abstract

**Background:**

The main aim of this study was to examine the perioperative results of reoperations and suggest novel surgical approaches. Based on a substantial number of robotic and laparoscopic nephron-sparing surgery (NSS), the authors aim to propose novel surgical strategies that offer practical recommendations to surgeons.

**Methods:**

Renal cell carcinoma patients with ipsilateral recurrent tumors, without evidence of metastasis, and who underwent primary NSS at our center between 2013 and 2023 were enrolled in this study, and all received the second time surgery. The authors conducted an analysis to evaluate perioperative outcomes and observed trends over a decade. Additionally, based on the findings from this study, the authors developed our surgical strategies.

**Results:**

In the past decade, our center has successfully conducted a total of 2546 surgeries for renal cell carcinoma, out of which this study includes 15 patients who met the specified criteria. For reoperation, robotic-assisted surgery was applied in five cases (33.3%), laparoscopic surgery in six cases (40%), and open surgeries in four cases (26.7%). While four (26.7%) patients underwent NSS while radical nephrectomy was performed on 11 patients (73.3%). The median operative time was 215 min (IQR: 135–300), and the median estimated blood loss was 50 ml (IQR: 50–100). The median length of postoperative hospitalization was 6 days (IQR: 5–9). Furthermore, there has been a yearly increase in the application of robotic-assisted NSS at our institution.

**Conclusion:**

Reoperation following the pNSS is a secure and effective surgical approach. The authors introduce novel surgical strategies for primary surgery and reoperation, which offer valuable insights to surgeons in the current study.

## Introduction

Highlights

- Our research indicates that the reoperation following primary nephron-sparing surgery is both safe and effective.
- Based on the extensive experience of our center in renal cell carcinoma, we propose innovative surgical strategies for both primary nephron-sparing surgery and reoperation.

Renal carcinoma accounts for 5% of all adult malignancies in males and 3% in females, positioning it as the seventh most prevalent neoplasm among men and the 10th among women. Roughly 80% of these instances are ascribed to renal cell carcinoma (RCC)^1,2^. Currently, surgery remains the established standard for treating localized RCC. Nephron-sparing surgery (NSS) or partial nephrectomy (PN), rather than radical nephrectomy (RN), is considered the optimal surgical approach for localized T1a-b tumors^3^.

With the rapid advancements in science and technology, the scope of NSS has expanded significantly, and large tumors are no longer regarded as insurmountable contraindications. In a multicenter cohort study conducted by Janssen *et al*.^4^, they demonstrated that NSS can be safely and effectively performed on renal tumors larger than 7 cm. In addition, when considering long-term overall survival (OS) and cancer-specific survival (CSS), NSS proves to be equally effective as RN.

However, recurrent tumor lesions, either locally or ectopically, may manifest in the remaining kidney following primary NSS (pNSS)^5^. Patients who underwent NSS with clinical stage T1 but progressed to pathological stage T3 exhibited a shorter relapse-free survival compared to those who underwent RN^6^. The incidence of ipsilateral RCC recurrence after pNSS has been reported to be ~5.6% in previous studies, and the presence of positive surgical margins (PSM) is associated with an increased risk of tumor recurrence^7^. Sorokin *et al*. conducted a study on 197 patients who underwent NSS at stage T1, among whom three experienced ipsilateral recurrences, resulting in a recurrence rate of 1.5%. Notably, the recurrences were observed to occur earlier in T1b cases compared to T1a cases^8^. The treatment of RCC encompasses surgery, pharmacotherapy, radiotherapy, chemotherapy, etc. Pharmacotherapy options include inhibitors targeting vascular endothelial growth factor receptor VEGFR, monoclonal antibodies against VEGF, and mammalian target of rapamycin (mTOR)^9^. Capitalizing on the triumphs of inhibitory antibodies that target the programmed cell death protein 1 (PD-1) PD-1 and cytotoxic T lymphocyte-associated antigen-4 (CTLA-4) CTLA-4 pathways, a plethora of novel immunotherapeutic interventions are currently undergoing clinical trials for the management of RCC. These include emerging Immune Checkpoint Inhibitors (ICIs), agonists of co-stimulatory pathways, engineered cytokines, modifiers of metabolic pathways, cellular treatments, and therapeutic vaccines^10^. The treatment options for recurrent RCC (rRCC) are progressively expanding; however, current research efforts primarily concentrate on metastasis investigation and recurrence factor prediction^11–13^, relevant study on reoperation for rRCC, particularly focusing on surgical techniques, remains scarce.

In the current retrospective study, we collected the perioperative data of rRCC patients who experienced a recurrence after the pNSS. Additionally, we examined the distribution of different surgical strategies and observed surgical trends at our high-volume center over the past decade. Significantly, based on a substantial number of robotic and laparoscopic NSS, as well as our expertise in reoperations for rRCC, we propose novel surgical perspectives that offer practical recommendations to surgeons performing pNSS while addressing challenges associated with potential reoperation for rRCC.

## Patients and methods

### Patients summary

We collected the patients who underwent reoperation for local rRCC after pNSS in our institution from 2013 to 2023. Local rRCC was defined as any recurrence in the ipsilateral kidney and retroperitoneum, with precise documentation of the anatomical site of recurrence regardless of its distance from the tumor excision site^14^. This study only enrolled patients who had undergone pNSS for ipsilateral RCC, without any distant metastasis. Patients with nonipsilateral rRCC and distant metastasis were excluded, nor were those lacking essential information.

### Measurements and outcomes

The patient demographics, characteristics of rRCC, and perioperative oncological data were meticulously documented using our institution’s HIS. All surgeries were conducted by highly skilled urological surgeons, with comprehensive descriptions available in previous research studies^15–18^. The operative time was calculated from the initial incision to the closure of the skin. The estimated blood loss was evaluated by subtracting the lavage fluid from the suction. The perioperative assessment was based on the American Society of Anesthesiologists (ASA) score^19^, Charlson Comorbidity Index (CCI)^20^, RENAL score^21^ and Clavien–Dindo classification (CDC)^22^.

### Follow-up

The urological surgeons recommend a follow-up protocol after reoperation, which typically includes abdominal and chest computed tomography (CT) scans every 6 months during the first 2 years postsurgery, followed by annual scans thereafter. Additionally, the estimated glomerular filtration rate (eGFR) and serum creatinine (Scr) measurements are required at the initial 3-month follow-up.

### Statistics

The analysis of continuous variables involved the utilization of medians and interquartile ranges (IQRs), while frequencies and proportions were employed for categorical variables. Statistical analyses were performed using IBM SPSS Statistic version 22 (IBM) and GraphPad Prism 8 (GraphPad Software Inc.). The work has been reported in line with the strengthening the reporting of cohort, cross-sectional, and case–control studies in surgery (STROCSS) criteria^23^.

## Results

In the past 10 years, our institution has successfully performed a cumulative number of 2546 surgeries for patients with renal carcinoma. Among these cases, 15 patients met our criteria, which means each of them received two times surgery in the ipsilateral renal, for the primary RCC and rRCC, we meticulously recorded their detailed clinical parameters of them during the second time inpatient admission (Table 1). For all the enrolled patients, males accounts for 93.3%, while the median age of first-time diagnosis is 58-year-old (IQR 54–65) and median BMI is 26.12 kg/m^2^ (IQR 21.80–27.76). The Charlson Comorbidity Index (CCI) ranged from 0 to 3, while the American Society of Anesthesiologists (ASA) score ranged from 2 to 3, and the renal score ranged from 5p to 9p. Among the patients, eight had a history of previous abdominal surgery (53.3%), which might increase the difficulty of surgery. The clinical T stage of rRCC varies between stages 1a and 2a. The median tumor diameter is about 3 cm (IQR: 2.5–4), whereas preoperative median creatinine serum level of 82 mg/dl (IQR: 75–95), and preoperative median eGFR is about 88 ml/min/1.73 m^2^ (IQR: 74–99). The tumor recurrence occurs over a span of 36 months (IQR: 20–55, Fig. 1A), with most recurrences observed within the previous tumor resection bed (86.7%, *n*=13). For the details of first-time surgery, robotic-assisted surgery was performed in 10 cases (66.7%) for primary RCC, followed by laparoscopic surgery in four cases (26.7%) and open surgery in one case (6.7%). None of the patients were detected with positive surgical margin after the pNSS.

**Table 1:** Basic clinical parameters for the enrolled patients.

Sex, no. (%)	
Male	14 (93.3)
Female	1 (6.7)
Age, median (IQR)	58 (54–65)
BMI, median (IQR)	26.12 (21.80–27.76)
CCI, no. (%)	
0	3 (20)
1	6 (40)
2	4 (26.7)
3	2 (13.3)
ASA score, no. (%)	
1	0 (0)
2	10 (66.7)
3	5 (33.3)
4	0 (0)
Previous history of abdominal surgery, no. (%)	8 (53.3)
Clinical T stage	
1a	11 (73.3)
1b	3 (20)
2a	1 (6.7)
Tumor side	
Left, *n* (%)	9 (60)
Right, *n* (%)	6 (40)
Tumor diameter, cm (IQR)	3 (2.5–4)
Renal score, no. (%)	
5p	4 (26.7)
6p	1 (6.7)
7a and 7p	2 (13.3)
8a and 8p	3 (20)
9a, 9h, and 9p	5 (33.3)
Preoperative creatinine serum level (mg/dl), median (IQR)	82 (75–95)
Preoperative eGFR (ml/min/1.73 m^2^), median (IQR)	88 (74–99)
Recurrence site, no. (%)	
Elsewhere in the ipsilateral kidney	2 (13.3)
Previous tumor resection bed	13 (86.7)
Time to recurrence (mo), median (IQR)	36 (20–55)
Surgical approach of primary RCC	
Robotic	10 (66.7)
Laparoscopic	4 (26.7)
Open	1 (6.7)
Positive surgical margin of primary RCC, *n* (%)	0 (0)

ASA, American Society of Anesthesiologists; CCI, Charlson Comorbidity Index; eGFR, estimated Glomerular Filtration Rate; IQR, interquartile range; RCC, renal cell carcinoma.

**Figure 1:**
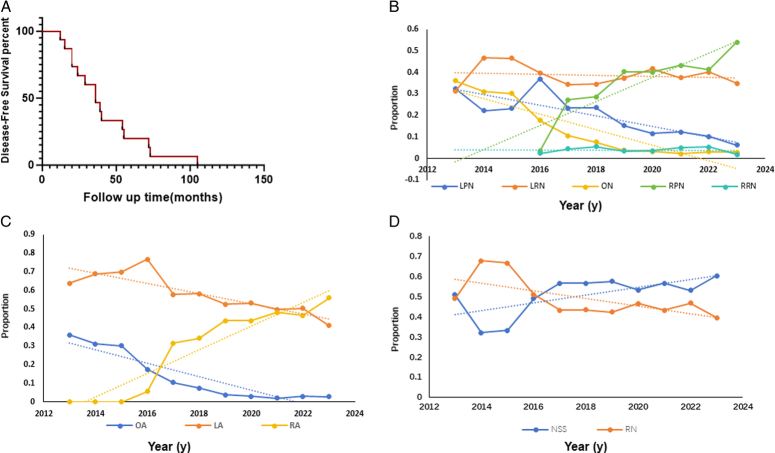
The trend of surgical approaches for renal cell carcinoma in our institution. (A) Disease-free survival (DFS) rates among RCC patients from pNSS to recurrence. (B) Trends in laparoscopic partial nephrectomy (LPN), laparoscopic radial nephrectomy (LRN), open nephrectomy (ON), robotic-assisted laparoscopic partial nephrectomy (RPN), and robotic-assisted laparoscopic radical nephrectomy (RRN) over the past decade. (C)Trends in open approach (OA), laparoscopic approach (LA) and robotic-assisted approach (LA) over the past decade. (D)Trends in nephron-sparing surgery (NSS) and radical nephrectomy (RN) over the past decade.

Taking into account each patient’s tumor location, tumor size, and physical condition, we performed different surgical approaches during the reoperation (Table 2), including robotic surgery in five cases (33.3%), laparoscopic surgery in six cases (40%), and open surgery in four cases (26.7%). Four patients (26.7%) underwent NSS, while RN was performed on 11 patients (73.3%). The median operative time was 215 min (IQR: 135–300), the median estimated blood loss was 50 ml (IQR: 50–100), and one patient required intraoperative blood transfusion due to significant blood loss and was subsequently transferred to the ICU. Postoperative outcomes are presented in Table 3. One patient received a blood transfusion due to postoperative low hemoglobin levels, while three patients required analgesics for postoperative pain and vomiting. The median postoperative creatinine serum level was 107 mg/dl (IQR: 91–119), and the median postoperative eGFR was 69 ml/min/1.73 m^2^ (IQR: 61–81). The median length of postoperative hospitalization was 6 days (IQR: 5–9), and the median duration until drainage tube removal was 5 days (IQR: 3–7). Among all cases, pT1a, pT1b, and pT3a stages were recorded in proportions of ~66.7, 20, and13.3% respectively. One patient exhibited stage pN1 with peripheral lymph node metastasis. No PSM were reported among any of the patients. For all 15 patients, the postoperative median follow-up time is about 24 months, ranging from 10 to 44 months. Twelve of them now still alive without any recurrence or metastasis, 1 of them developed tongue metastasis after reoperation, and the other two missed.

**Table 2:** Surgery detail and intraoperative outcomes for the reoperation.

Surgical approach, no. (%)	
Robotic	5 (33.3)
Laparoscopic	6 (40)
Open	4 (26.7)
Partial or total nephrectomy, no. (%)	
Partial	4 (26.7)
Total	11 (73.3)
Operative time (min), median (IQR)	215 (135–300)
Estimated blood loss, median (IQR)	50 (50–100)
Intraoperative blood transfusion, no. (%)	1 (6.7)
Radical nephrectomy conversions, no. (%)	0 (0)
Surgical approach conversions, no. (%)	0 (0)
Intraoperative complications, no. (%)	1 (6.7)

IQR, interquartile range.

**Table 3:** Postoperative outcomes of the enrolled 15 patients.

Postoperative blood transfusion, no. (%)	1 (6.7)
Clavien–Dindo, no. (%)	
1	3 (20)
2	1 (6.7)
3	0 (0)
4	0 (0)
Postoperative creatinine serum level (mg/dl), median (IQR)	107 (91–119)
Postoperative eGFR (ml/min/1.73 m^2^), median (IQR)	69 (61–81)
Length of postoperative hospitalization (d), median (IQR)	6 (5–9)
Drainage tube removal time (d), median (IQR)	5 (3–7)
Pathological type, no. (%)	
Clear cell carcinoma	13 (86.7)
Papillary cell carcinoma	1 (6.7)
Chromophobe cell carcinoma	1 (6.7)
Positive surgical margins, no. (%)	0 (0)
pT stage, no. (%)	
1a	10 (66.7)
1b	3 (20)
3a	2 (13.3)
pN stage, no. (%)	
N0	14 (93.3)
N1	1 (6.7)
Follow-up length (month), median (IQR)	24 (10–44)
Survival status, no. (%)	
Alive without metastasis	12 (80)
Alive with metastasis	1 (6.7)
Loss to follow-up	2 (13.3)
Postoperative CKD, no. (%)	0 (0)
Re- recurrence, no. (%)	0 (0)

CKD, chronic kidney disease; IQR, interquartile range.

We also focused on the tumor pathological types among both two times surgery. After reoperation, clear cell renal cell carcinoma (ccRCC) was reported that accounted for the majority of cases (13/15, 86.7%), while all the patients showed the negative surgical margins. One patient was diagnosed with ccRCC after the pNSS, but chromophobe cell carcinoma after the reoperation. Unfortunately, the WHO/ISUP grading system is not applicable for chromophobe cell carcinoma, thus impeding the assessment of disease progression in this case. Moreover, accurate pathological evaluation of two patients who underwent their pNSS at an external medical facility was unattainable. The remaining patients exhibited consistent pathological types in both two times surgery. Notably, three patients demonstrated an upgrade of WHO/ISUP grade after reoperation compared to the first-time surgery (Table 4).

**Table 4:** The postoperative characteristics of pNSS and reoperation.

	pNSS			Reoperation		
No.	Pathology	WHO/ISUP	Invasion of perirenal adipose tissue	Pathology	WHO/ISUP	Invasion of perirenal adipose tissue
**1**	Clear cell RCC	1	No	Clear cell RCC	2	No
**2**	Clear cell RCC	3	No	Clear cell RCC	3	No
**3**	Clear cell RCC	3	Yes	Chromophobe RCC	N/A	Yes
**4**	Clear cell RCC	2	No	Clear cell RCC	2	No
**5**	Clear cell RCC	2	No	Clear cell RCC	3	No
**6**	Clear cell RCC	2	Yes	Clear cell RCC	2	No
**7**	Clear cell RCC	1	No	Clear cell RCC	2	No
**8**	Papillary RCC	2	No	Papillary RCC	2	No
**9**	Clear cell RCC	2	No	Clear cell RCC	2	No
**10**	Clear cell RCC	N/A	N/A	Clear cell RCC	2	No
**11**	Clear cell RCC	N/A	N/A	Clear cell RCC	1	No
**12**	Clear cell RCC	2	No	Clear cell RCC	2	Yes
**13**	Clear cell RCC	2	No	Clear cell RCC	2	No
**14**	Clear cell RCC	3	Yes	Clear cell RCC	3	No
**15**	Clear cell RCC	2	No	Clear cell RCC	2	No

Case 3 N/A: WHO/ISUP grading system is not applicable for chromophobe cell carcinoma; Case10 and 11 N/A: two patients underwent pNSS at an external medical facility, whose detailed pathology could not be queried; pNSS, primary nephron-sparing surgery; RCC, renal cell carcinoma.

In addition, the use of robotic-assisted surgery has consistently grown each year in our high-volume urological center, particularly the robotic-assisted laparoscopic partial nephrectomy (RPN). Conversely, there has been a gradual decrease observed over time in the utilization of laparoscopic partial nephrectomy (LPN), laparoscopic radial nephrectomy (LRN), and open nephrectomy (ON) (Fig. 1B). The proportion of surgeries performed with robotic assistance is steadily increasing while both laparoscopic and open surgeries are declining (Fig. 1C). Similarly, there is a progressive rise in the percentage of NSS, which have surpassed RN as the primary choice (Fig. 1D).

## Discussion

With the advancement of science and technology, coupled with the continuous enhancement of surgeons’ expertise, there is a projected substantial growth in the utilization of robotic-assisted laparoscopy and NSS. Consequently, the likelihood of recurrence following pNSS will be further amplified. Hence, it becomes imperative to explore the optimal surgical techniques for rRCC after pNSS and even pNSS. Our research group has dedicated efforts towards enhancing and innovating surgical approaches and equipment, optimizing minimally invasive surgeries such as NSS. We have pioneered the ‘Combined Retroperitoneoscopic and Transperitoneoscopic Accesses’ – robot-assisted laparoscopic NSS globally, as well as developed the first 5G wireless intelligent laparoscopy systems all over the world, which have been actually applied to clinical surgery, providing great convenience for surgeons^24,25^. In this study, we creatively present our surgical concepts and techniques based on a retrospective analysis of patients treated at a high-volume urological center.

However, limited research has been conducted on reoperation for rRCC. Fabrizio *et al*. emphasized the safety and feasibility of redoing NSS for local rRCCs following pNSS. Due to the presence of perirenal adhesions resulting from the pNSS, the researchers recommended reoperation without clamping the renal hilum whenever possible. Additionally, they suggested utilizing intraoperative ultrasound and hybrid enucleative-enucleoresective dissection^14^. Other studies also suggested that robotic-assisted NSS after pNSS is safe and efficacious^26–28^. Some scholars have also proposed that RN was adapted to metastatic and recurrent patients^5^. Consequently, the existing literature predominantly emphasizes the validation of diverse reoperations in terms of safety and feasibility, rather than providing urological surgeons with valuable insights into surgical techniques.

The occurrence of adhesion in the surrounding area following surgery is widely acknowledged, thereby augmenting the intricacy of subsequent operations and presenting substantial challenges for surgeons. The CT images should be meticulously analyzed prior to the reoperation in order to precisely determine the exact location of the pNSS, thereby facilitating its avoidance during the subsequent surgery. Similarly, it is imperative to avoid the site of renal artery occlusion encountered during the pNSS or any other methods of arterial occlusion, such as percutaneous balloon occlusion of the renal artery. In terms of tumor resection and wound suture, the surgical range should be expanded beyond that of the pNSS, and the intraoperative ultrasound mentioned above is needed. When encountering the Hem-o-lok of the pNSS, it is recommended to shift the surgical resection surface outwards. Additionally, in reoperation, the dissection plane should be directed away from the pNSS wound, and more tissue should be excised to reduce the occurrence of PSM.

In addition, it is important to note that we present the surgical techniques of the pNSS by elucidating the challenges encountered during the reoperation. To the best of our knowledge, this is the first time it has been proposed. During the pNSS, it is advisable to minimize extensive dissection of the parenchyma and artery of the kidney, avoid opening the renal artery sheath, and selective renal artery occlusion is preferred. When performing tumor resection and wound suturing, it is crucial to restrict excessive adipose dissection surrounding the tumor while maximizing the preservation of normal renal parenchyma during surgical excision. Additionally, attention should be given to suture margins in order to minimize the use of Hem-o-lok during suturing or choose a smaller Hem-o-lock to reduce inflammatory wrapping. Moreover, caution should be exercised in utilizing perirenal adipose tissue for wound coverage upon completion of suturing. If these skills are given due attention in the pNSS, we firmly believe that the proportion of NSS in the reoperation can be further augmented.

The analysis of 10 years of surgery in our high-volume urologic center indicates that minimally invasive surgery, particularly robot-assisted laparoscopic surgery and NSS, will emerge as the prevailing trend in the future. This finding is consistent with previous studies^29–31^. Compared with RN, the postoperative recurrence rate of NSS is still controversial^6,32,33^. Therefore, the recurrence after NSS should not be neglected. With the wide application of robotic-assisted NSS, the recurrence of RCC and even second recurrence may also increase. It is of utmost significance to ensure optimal surgical visualization and operating area for potential reoperation or even subsequent re-reoperations in NSS.

Among the 2546 patients, 1238 underwent NSS, and only 15 were screened for reoperation, resulting in a recurrence rate of ~1.2%. Due to the limited sample size, it was not possible to identify risk factors associated with clinical characteristics of patients experiencing local recurrence. However, we did summarize and present the clinical characteristics of these patients, including sex, age, BMI, CCI, ASA score, previous history of abdominal surgery, surgical approach, tumor diameter, and location. The 2022 edition of the European Association of Urology Guidelines for RCC states that renal cancer is more prevalent in men than women at a ratio of 1.5:1. Among our cohort of 15 patients, 14 were male and only one was female; thus suggesting a preliminary observation that men may have a higher recurrence rate than women. Additionally, smoking, obesity, and hypertension are significant risk factors^28^. It is important to emphasize that regular postsurgical follow-up examinations should be conducted for both primary and re-NSS in order to achieve early diagnosis and prompt treatment if any tumor recurrence occurs.

Limitations of our research should be emphasized. Firstly, our study is a retrospective analysis with a small sample size and restricted follow-up duration. The second limitation was the obvious selection bias, as only patients who met the indication underwent surgery. Finally, most patients underwent RN for recurrence in the early stage, resulting in a reduced proportion of NSS in our study. Multicenter studies should be undertaken to record patient characteristics and perioperative outcomes more accurately. Nevertheless, we have presented a series that demonstrates both safety and feasibility. Based on these premises, we propose our innovative perspective to offer a valuable reference for urologists. Undoubtedly, future endeavors should focus on conducting prospective, multicenter, and large-scale randomized controlled trials.

## Conclusions

Our findings indicate that reoperation following pNSS is both safe and feasible, regardless of whether a re-NSS or re-RN was employed. It is worth noting that our study, which encompassed a substantial number of cases to innovatively summarize surgical strategies for primary surgery and reoperation, which offers valuable insights to surgeons. We believe that these novel surgical strategies may hold great significance in clinical practice.

## Ethical approval

This study received approval from the Ethics Committee at the First Affiliated Hospital of Anhui Medical University (PJ2024-01-44) and complied with the Declaration of Helsinki.

## Consent

There is wavier of consent to publication from institutional review board of the First Affiliated Hospital of Anhui Medical University as all data were retrospectively collected with information anonymized. The submission does not include information or images that may identify any person.

## Source of funding

This research was supported by the Postgraduate Innovation Research and Practice Program of Anhui Medical University (YJS20230112), the Anhui Provincial University Excellent Scientific Research and Innovation Team Project (2022AH010071), and the Anhui Provincial Central Government Guides Local Science and Technology Development Project-Anhui Clinical Research Center of Urology Disease (2019b07030004).

## Author contribution

C.L.: study concepts and study design; W.M. and W.X.: data acquisition; J.M., L.C., S.T., C.Y., J.C., and H.S.: quality control of data and algorithms; J.M., W.M., W.X.: data analysis and interpretation, manuscript preparation, and manuscript editing. All authors approved the publication of the manuscript.

## Conflicts of interest disclosure

The authors have no conflicts of interest.

## Research registration unique identifying number (UIN)

We register this study in ClinicalTrials.gov, with the Identifiers: NCT06317207; Unique Protocol ID: PJ2024-01-44.

## Guarantor

Chaozhao Liang.

## Data availability statement

The data used during the current study are available from the authors upon reasonable request and with permission of Department of Medical Administration, National Health Commission of the People’s Republic of China.

## Provenance and peer review

This paper is not invited.
